# Comprehensive Assessment of Protein and Excipient Stability in Biopharmaceutical Formulations Using ^1^H NMR Spectroscopy

**DOI:** 10.1021/acsptsci.0c00188

**Published:** 2020-12-16

**Authors:** Jack E. Bramham, Adrian Podmore, Stephanie A. Davies, Alexander P. Golovanov

**Affiliations:** † Manchester Institute of Biotechnology and School of Chemistry, Faculty of Science and Engineering, The University of Manchester, Manchester M1 7DN, U.K.; ‡ Dosage Form Design & Development, BioPharmaceuticals Development, R&D, AstraZeneca, Cambridge CB21 6GH, U.K.

**Keywords:** biopharmaceutical formulation, NMR spectroscopy, excipients, fragmentation, aggregation

## Abstract

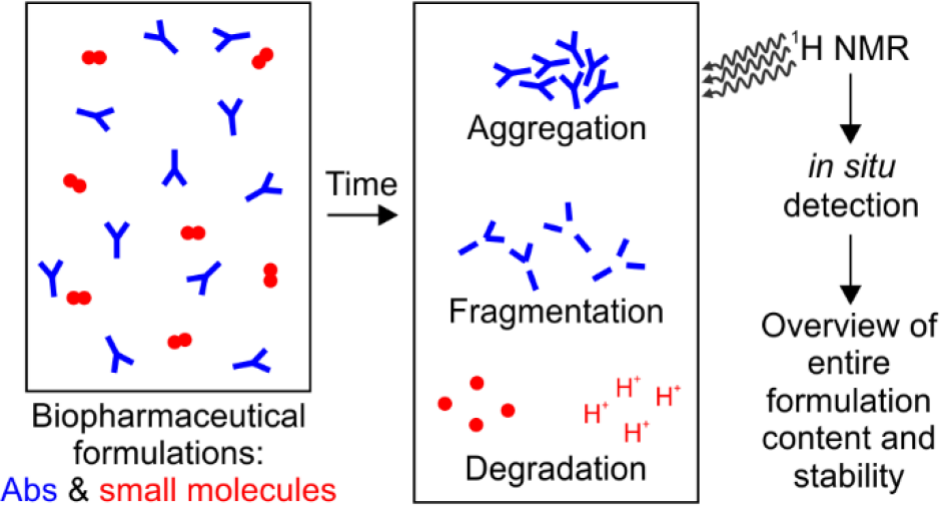

Biopharmaceutical proteins are important drug therapies in the treatment of a range of diseases. Proteins, such as antibodies (Abs) and peptides, are prone to chemical and physical degradation, particularly at the high concentrations currently sought for subcutaneous injections, and so formulation conditions, including buffers and excipients, must be optimized to minimize such instabilities. Therefore, both the protein and small molecule content of biopharmaceutical formulations and their stability are critical to a treatment’s success. However, assessing all aspects of protein and small molecule stability currently requires a large number of analytical techniques, most of which involve sample dilution or other manipulations which may themselves distort sample behavior. Here, we demonstrate the application of ^1^H nuclear magnetic resonance (NMR) spectroscopy to study both protein and small molecule content and stability *in situ* in high-concentration (100 mg/mL) Ab formulations. We show that protein degradation (aggregation or fragmentation) can be detected as changes in 1D ^1^H NMR signal intensity, while apparent relaxation rates are specifically sensitive to Ab fragmentation. Simultaneously, relaxation-filtered spectra reveal the presence and degradation of small molecule components such as excipients, as well as changes in general solution properties, such as pH. ^1^H NMR spectroscopy can thus provide a holistic overview of biopharmaceutical formulation content and stability, providing a preliminary characterization of degradation and acting as a triaging step to guide further analytical techniques.

## Introduction

Biopharmaceutical antibodies (Abs), such as monoclonal antibodies (mAbs) and, more recently, bispecific antibodies (BsAbs), are increasingly important therapies in the treatment of a wide range of diseases, including cancer, arthritis, and diabetes. Within the biopharmaceutical industry, there is considerable interest in the development of high-concentration (>100 mg/mL) protein formulations to enable subcutaneous administration of the lowest possible volume injection,^1,2^ potentially by the patient themselves.^3^ Such administration strategies result in lower treatment costs and better patient experience, particularly in the treatment of chronic conditions such as autoimmune disorders.^4^ However, high protein concentrations are associated with increased levels of physical instabilities, such as self-association,^5,6^ aggregation,^7^ and liquid–liquid phase separation (LLPS),^8−10^ in addition to chemical degradation, such as fragmentation^11,12^ and oxidation.

To ensure therapeutic proteins remain stable and therefore safe and efficacious at high concentrations, formulation conditions, such as buffers, pH, ionic strength, and small molecule excipients, must be optimized.^13,14^ In this regard, the long-term stabilities of both the protein molecules themselves and the small molecule formulation components are important. Degradation of the small molecule components required for protein stability,^15,16^ or their reaction with proteins,^17,18^ may subsequently lead to protein instabilities. Optimisation of formulation conditions, and continued assessment of formulation stability, requires analytical techniques capable of assessing both protein and small molecule content and behavior, ideally *in situ* in intact formulations. However, in practice, a wide range of techniques is deployed,^19−21^ and these techniques typically require manipulation of high-concentration formulations, such as dilution, addition of a probe molecule, or salt removal, potentially leading to changes in protein and small molecule structure and behavior.

Nuclear magnetic resonance (NMR) spectroscopy is a powerful biophysical technique, which can be applied *in situ* at high concentration without sample dilution. The range of NMR experiments, from saturation transfer for detection of protein-excipient interactions^22,23^ to diffusion and relaxation assessment of mAb solution behavior,^24,25^ and fingerprinting of higher-order structures of mAbs and biosimilars^26−28^ mean that NMR can be used to study a wide range of biopharmaceutical problems. In formulation studies, NMR has been used to characterize the presence of residual small molecule contaminants from bioprocessing^29,30^ and to quantify small molecule levels,^31^ while low resolution benchtop spectroscopy has been applied to study mAb degradation based on broad changes in the relaxation rate of the water signal.^32,33^ For complex formulations, there may be advantages in observing multiple parameters to characterize multiple degradation pathways, and, in principle, high-resolution ^1^H NMR allows observation of all proton containing species, including proteins and small molecules.

Here, we explore the use of high-resolution ^1^H NMR spectroscopy to report on the content and behavior of both small molecule and Ab protein components simultaneously in model formulations. For three high-concentration Abs (100 mg/mL) stored under stressed stability conditions (40 °C), we demonstrate that complex ^1^H NMR spectra of Ab solutions can be separated into small molecule and protein regions by the application of a simple transverse relaxation (T_2_) filter. Having spectroscopically separated protein and small molecule components, we show that the signal intensities and apparent relaxation rates of Abs can be used to monitor protein stability and the occurrence of degradation, such as aggregation and fragmentation, over a 12-week period. NMR observations are compared with a standard size-exclusion chromatography analysis. Additionally, the appearance, intensity, and chemical shift of small molecule components can be simultaneously used to study the presence and degradation of excipients themselves, as well as changes in general solution properties, such as pH. Through the use of sealed NMR tubes with coaxial inserts, these assessments are observed *in situ* at high concentration without sample dilution or manipulation. We demonstrate that ^1^H NMR spectroscopy is a suitable orthogonal technique to provide a comprehensive overview of formulation content and stability and can act as a triaging step to guide further detailed analysis.

## Materials and Methods

### Sample Preparation

Two mAbs and a BsAb were supplied by AstraZeneca: COE-03 (IgG1, MW 144.8 kDa, pI 8.44), COE-07 (bispecific IgG1, MW 196.7 kDa, pI 8.0), and COE-19 (IgG1, MW 148 kDa, pI 7.4–7.9). All Abs were dialyzed (six buffer exchanges over 3 days) into 20 mM phosphate buffer, pH 6.5 (sodium phosphate dibasic (Na_2_HPO_4_) and sodium phosphate monobasic (NaH_2_PO_4_) (both Sigma-Aldrich)), with 200 mM NaCl (Fisher) in GeBAflex-Maxi-tubes (MWCO 8 kDa, Generon, rinsed with 20% ethanol and then distilled water). Small molecules from the original formulations remaining after this extensive dialysis, and the phosphate buffer with NaCl, were treated as the final model formulations. Sample concentration was conducted in Vivaspin 20 centrifugal concentrators (MWCO 30 kDa, Sartorius), with final solutions filtered using 0.22 μm filters (PVDF, Merck Millipore). Concentration measurements were based on absorbance at 280 nm (A280) using known extinction coefficients and a NanoDrop spectrophotometer (Thermo Scientific).

All samples were prepared to 100 mg/mL protein concentration, with 0.05% sodium azide (Fisher) added to prevent bacterial growth. Samples for NMR spectroscopy (400 μL) were prepared in triplicate and placed in 5 mm borosilicate glass NMR tubes (Wilmad-LabGlass), with a coaxial insert (50 mm stem height, Wilmad-LabGlass) containing 60 μL of ^2^H_2_O (Sigma-Aldrich) to provide a spectrometer lock without sample dilution or change in formulation. Samples (one per time point) for HPSEC were placed in borosilicate glass vials (Sigma-Aldrich, 1 mL). All samples were sealed with an appropriate cap and Parafilm wrap (Cole-Parmer) and stored upright at 40 °C in a Heratherm compact incubator (Thermo Scientific, uniformity ±1.2 °C, stability ±0.2 °C). One non-NMR sample per Ab was frozen at each time point for analysis at a later date.

### NMR Spectroscopy

NMR experiments were acquired at 40 °C using a Bruker 800 MHz Avance III spectrometer equipped with a 5 mm TCI cryoprobe and variable temperature control unit, with temperature calibrated against a standard methanol sample and verified with an external thermocouple placed in a sample tube.

^1^H 1D spectra were recorded using WATERGATE water suppression (p3919gp Bruker pulse program), with this water suppression also used in relaxation experiments. Longitudinal relaxation rates (*R*_1_) were measured using the standard Bruker inversion recovery sequence (t1ir), with 10 recovery delays ranging from 1 ms to 3 s. Transverse relaxation rates (*R*_2_) were measured using a Carr–Purcell–Meiboom–Gill (CMPG) sequence, with temperature compensation to ensure equal sample heating during the CPMG acquisition and a fixed echo time of 3.6 ms. T_2_-filtered spectra were extracted from the CPMG data, with 32 echoes producing a 116 ms relaxation filter.

Spectra were processed and analyzed using Topspin 4.0 (Bruker). Apparent ^1^H longitudinal and transverse relaxation rates at spectral points (0.05 ppm intervals) across the spectral width were calculated in Dynamics Center 2.6 (Bruker). Relaxation rates were fitted to single component models, with two or more component models not significantly improving fitting. The processed data were plotted in GraphPad Prism 6.0.

### High Performance Size Exclusion Chromatography

Analysis of mAb and BsAb monomeric, aggregate, and fragment species was performed using high performance size exclusion chromatography (HPSEC). This was performed using an Agilent 1200 system with a TSKgel SW_XL_ column (30 cm × 7.8 mm, 5 μm particle size, Tosoh Bioscience). Samples were diluted to 10 mg/mL and 0.45 μm filtered prior to assessment with centrifugal filters (Ultrafree-MC-HV, Merck Millipore). Twenty-five μL was injected each time, and the system was run at 1.0 mL/min, with a mobile phase of 0.1 M Na_2_HPO_4_, 0.1 M Na_2_SO_4_, pH 6.8. The absorbance wavelength for detection was set at 280 nm. Chromatograms were analyzed in ChemStation (Agilent).

## Results

### Initial NMR Characterization of Formulation Content

**Figure 1:**
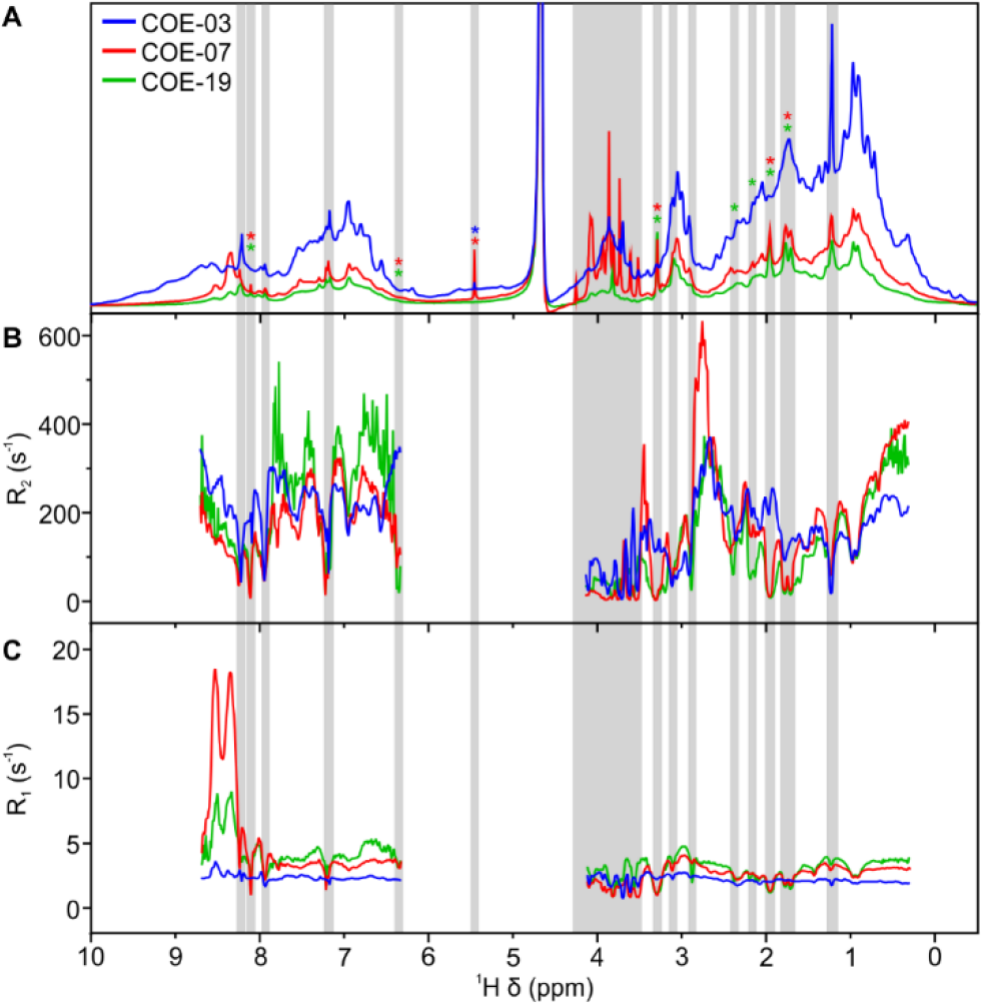
Initial NMR spectra and relaxation rates of the three Abs recorded at 40 °C. (A) ^1^H NMR spectra overlay of COE-03 (blue), COE-07 (red), and COE-19 (green). Apparent *R*_2_ (B) and *R*_1_ (C) were measured for each spectral point. Mean rates were from triplicate samples. Gray areas include signals arising from small molecule components. Colored asterisks indicate small molecules present in specified Ab samples only. Blank regions in apparent relaxation spectra were excluded due to low signal and large measurement errors. Residual water signal at 4.7 ppm.

Protein formulations are routinely stored at elevated temperatures to trigger degradation and infer long-term formulation stability. Here, we used this stressed stability approach to explore how ^1^H NMR spectroscopy can be used to study the content and stability of nonlabeled Abs samples, such as those obtained from mammalian production pipelines which do not permit easy isotope labeling. First, the initial baseline ^1^H NMR spectra and parameters were recorded for each of the three Ab formulations at 40 °C (at time *t* = 0). The acquired NMR spectra represent a complex mixture of overlapping protein and small molecule signals (Figure 1A). Despite this overlap, some spectral regions are clearly dominated by sharper, more intense signals arising from small molecules, which are also often present in much larger concentrations. As measuring individual proton longitudinal (*R*_1_) or transverse (*R*_2_) relaxation rates is impossible, we measured the apparent relaxation rates for each point in the spectra at 0.05 ppm intervals, thus providing a characteristic relaxation profile for each formulation (Figure 1B,C). As large and small molecules have significantly different tumbling rates, their *R*_2_’s are also significantly different. Factoring that excipients and small molecules have an *R*_2_ < 75 s^–1^, the spectra can be classified into regions dominated by Ab signals (shown in white) and small molecules (shown in gray).

In the protein-dominated regions of ^1^H spectra (Figure 1A), the three Abs display markedly different signal intensities despite identical protein concentrations and similar formulations. COE-07 and COE-19 signals generally exhibited faster relaxation rates than COE-03 (Figure 1B,C). These spectra and parameters indicate that COE-07 and COE-19 exhibit greater self-association than COE-03, in agreement with previous observations of the three Abs’ behavior.^34^ Noticeably, despite the differences elsewhere, the characteristic Ab methyl signals at 0.9–1.0 ppm result in similar *R*_1_ and *R*_2_ values for all three Abs, suggesting this invariant spectral region represents a flexible structural feature common to all Abs tested. Additionally, *R*_1_ values in the protein spectral region around 8.5 ppm appear acutely sensitive to differences between the Abs.

The presence of small molecule components (shaded gray in Figure 1) is easily identified by their slow *R*_2_, and their separated spectra is most conveniently obtained by running T_2_-filtered experiments^30^ (Figure S1). These T_2_-filtered spectra reveal the presence of residual components from the original formulations which were not completely removed by multiple rounds of dialysis during sample preparation–histidine in all three Abs, sucrose in COE-03 and COE-07, and arginine in COE-07 and COE-19. Additionally, trace ethanol was present in all Ab solutions, likely carried over from washing dialysis membranes before use. Protein translational diffusion (*D*_L_), in principle, may also report on molecular size;^25^ however, for such concentrated Ab solutions, the quality of ^1^H DOSY spectra was very poor for Ab signals (data not shown) due to particularly fast relaxation and slow diffusion. Therefore, relaxation profiling using *R*_1_ and *R*_2_ values provides a sensitive alternative for characterizing highly concentrated Ab formulations.

### Changes in Ab 1D ^1^H NMR Spectra upon Accelerated Stability Storage

**Figure 2:**
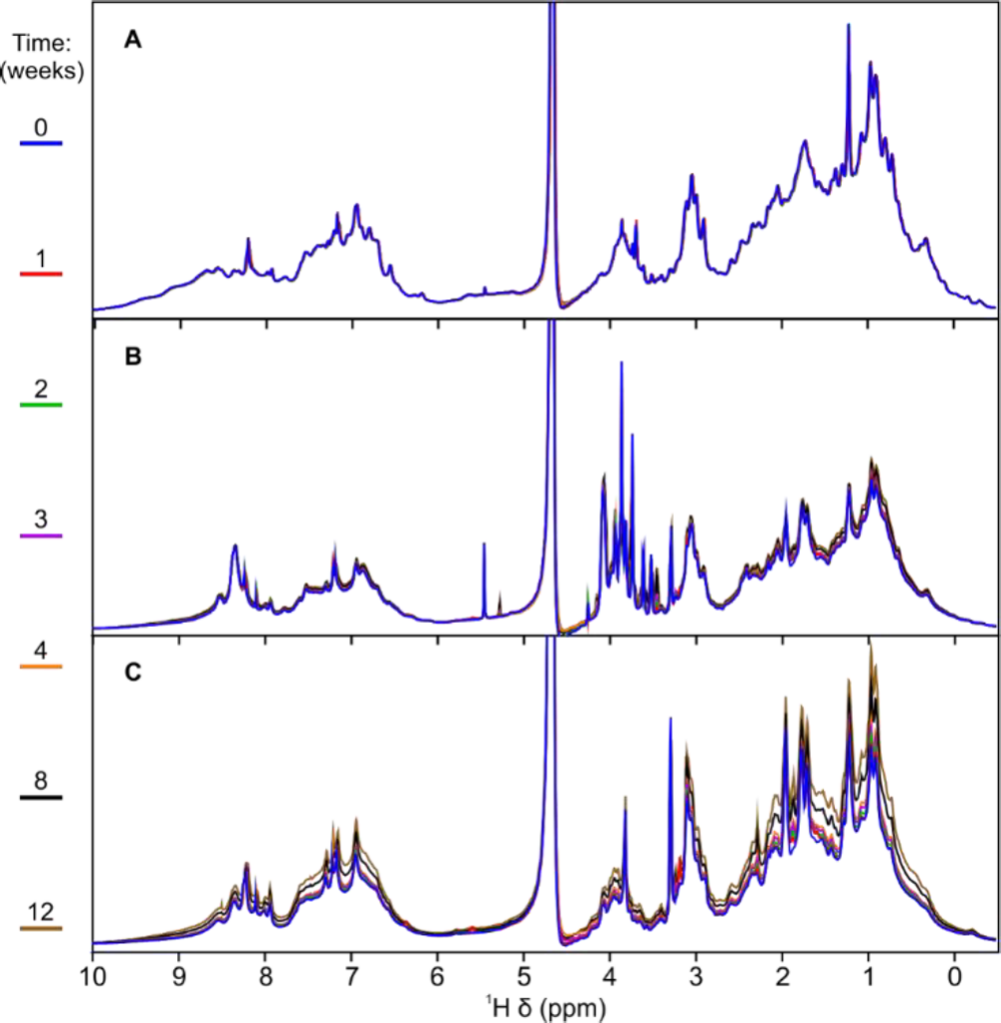
Changes in ^1^H NMR spectra during accelerated stability storage at 40 °C over time. (A) COE-03, (B) COE-07, and (C) COE-19. Spectra for COE-07 and COE-19 are scaled up for clarity 2-fold and 3-fold, respectively, compared to COE-03 spectra.

Having acquired baseline spectra and parameters for the Ab and small molecule components at the initial time point, NMR experiments were subsequently acquired after 1, 2, 3, 4, 8, and 12 weeks storage at 40 °C for the same sealed samples. 1D ^1^H NMR spectra represent the simplest and fastest acquired experiments, so they may provide the easiest assessment of Ab stability. The ^1^H NMR spectra of COE-07 and COE-19 exhibit increases in signal intensity with time (Figure 2), indicating protein degradation. Noticeably, the upscaled spectra of these Abs retain the same overall shape as the initial spectra. As the amount of material present in the sample remains the same, the broad increase in signal intensity across the spectra for COE-07 and COE-19 suggests predominantly fragmentation occurring, resulting in smaller freely tumbling Ab domains. These retain the same general spectral profile as the intact Ab due to the same domain fold but have higher apparent signal intensity due to their smaller molecular weight and hence faster rotational correlation time (τ_c_). Conversely, COE-03 spectra were largely unchanged, even after 12 weeks storage at 40 °C.

We have previously shown that protein aggregation results in decreases in observed signal intensities as slower-tumbling or NMR-invisible species are formed.^35^ Therefore, the predominant changes in NMR signal intensity for a given sample reveal the predominant underlying Ab degradation process, with increasing and decreasing intensities reflecting fragmentation and aggregation, respectively. In a more complex scenario, if both fragmentation and aggregation occur simultaneously, then it may be envisaged that opposing changes in intensity may largely balance each other (see Figure S2 for illustrative modeling). As we will show, this is the case for COE-03, where no significant change in intensity is observed (Figure 2A). In such a situation, additional spectral considerations need to be taken into account to correctly interpret whether no degradation has occurred, or whether fragmentation and aggregation have occurred simultaneously.

### Changes in Protein Apparent Relaxation Rates during Accelerated Stability Storage

**Figure 3:**
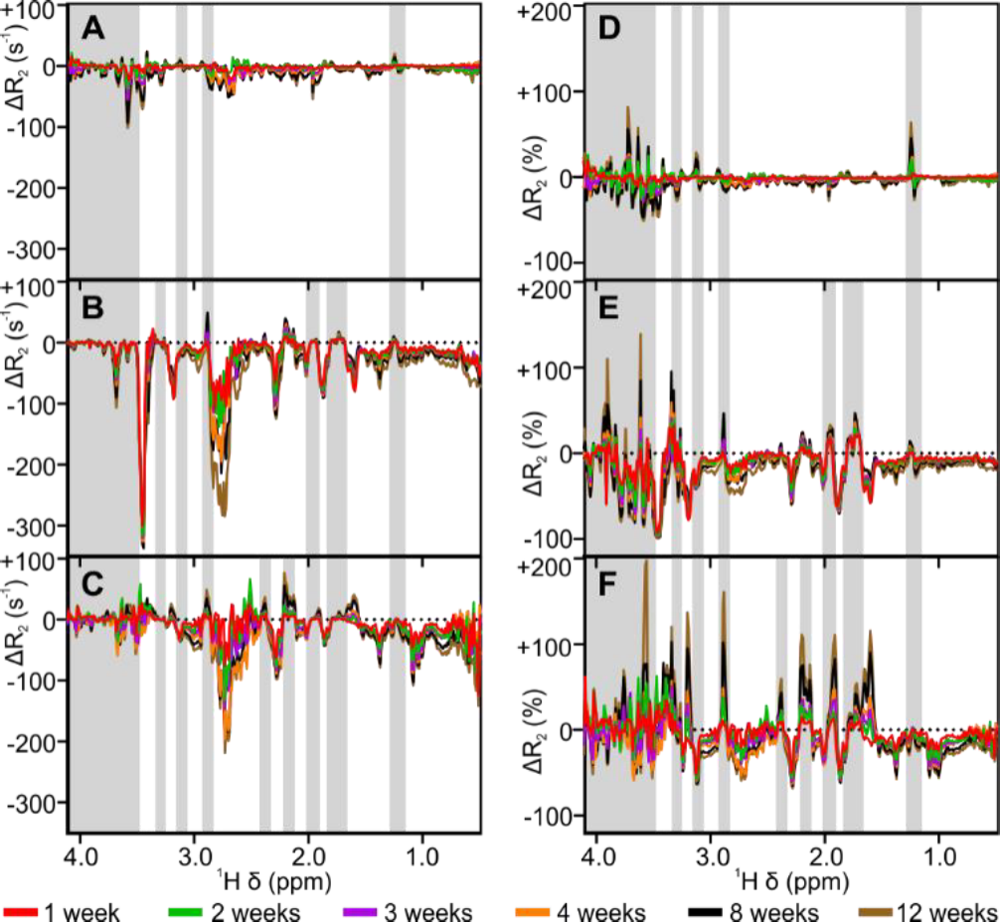
Changes in ^1^H *R*_2_ during accelerated stability storage. Absolute change (s^–1^) in *R*_2_ for (A) COE-03, (B) COE-07, and (C) COE-19. Change in *R*_2_ relative to the initial value at time *t* = 0 (%) for (D) COE-03, (E) COE-07, and (F) COE-19. Gray areas highlight regions of spectra containing small molecule signals. The dotted line denotes a baseline with no change.

Given the potential complex behavior of 1D ^1^H NMR spectra in response to degradation, we next considered changes in apparent relaxation rates. Large protein aggregates, such as those resulting from >150 kDa Abs, with slow τ_c_ and rapid *R*_2_ are likely largely “NMR-invisible” and, as such, make a negligible contribution to the measured apparent rates. Conversely, small protein species with faster tumbling are expected to contribute more prominently. For all three Abs, the apparent *R*_2_ values for protein-dominated spectral regions show a tendency to decrease with storage time (Figure 3). The reductions in Ab *R*_2_ with time are larger for COE-07 and COE-19 than for COE-03, both for absolute values (Figure 3A–C) and for relative values expressed as a percentage of the original *R*_2_ values (Figure 3D–F). As the *R*_2_ rate for a large protein is roughly proportional to the molecular size, the observed decreases in *R*_2_ are consistent with an average decrease in the molecular size of the observed species, i.e., the occurrence of protein fragmentation. In this respect, the changes in absolute *R*_2_ values for the spectral region around 2.8 ppm appear to be particularly sensitive to fragmentation given its relatively high initial *R*_2_ for all three Abs. The behavior in spectral regions dominated by small molecule signals is more complex and cannot be interpreted based solely by *R*_2_ values. Our further analysis (below) reveals chemical changes occurring for these formulation components.

**Figure 4:**
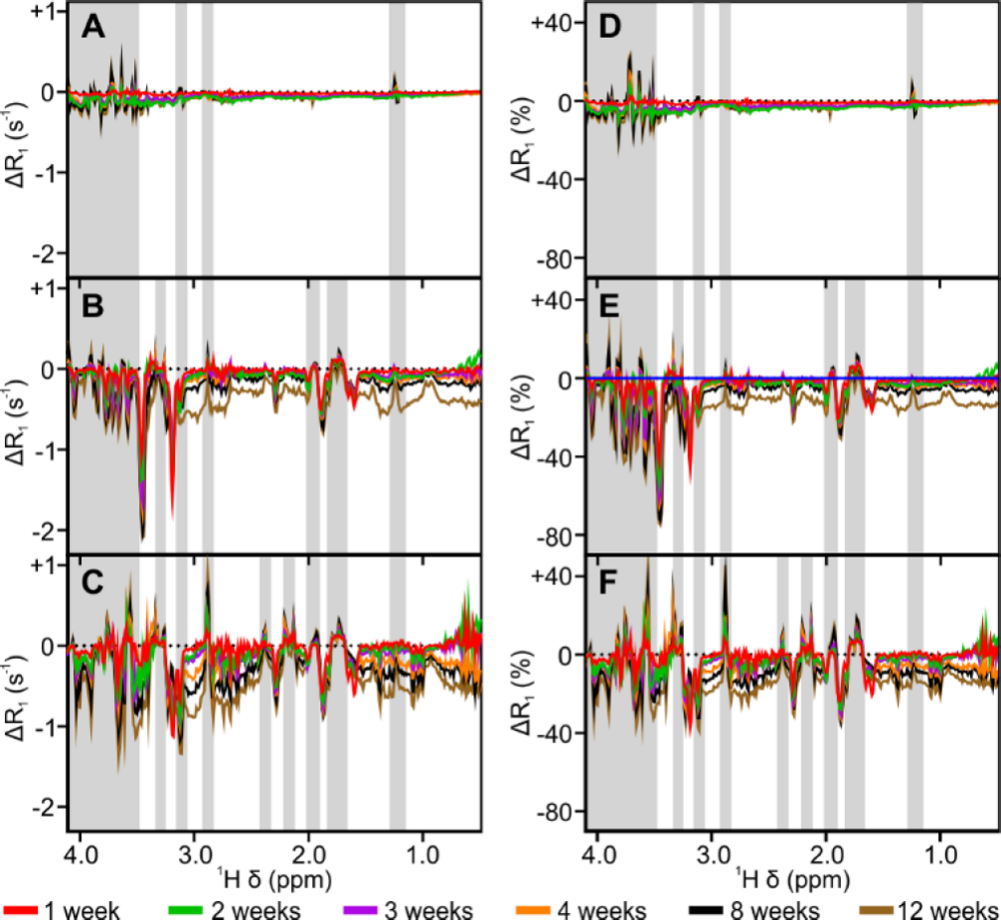
Changes in ^1^H *R*_1_ during accelerated stability storage. Absolute change (s^–1^) in *R*_1_ for (A) COE-03, (B) COE-07, and (C) COE-19. Change in *R*_1_ relative to the initial value at time *t* = 0 (%) for (D) COE-03, (E) COE-07, and (F) COE-19. Gray areas highlight regions of spectra containing small molecule signals. The dotted line denotes a baseline with no change.

*R*_1_ values are more complex to interpret than *R*_2_ in terms of molecular tumbling rates, given the V-shaped relationship between τ_c_ and longitudinal relaxation. Here, all Ab signals exhibit reductions in *R*_1_ rates with time which are fairly linear across the breadth of the Ab NMR spectra (Figure 4). Again, COE-07 and COE-19 exhibit larger decreases in the relaxation rate than COE-03 for both absolute (Figure 4A–C) and relative values (Figure 4D–F). Together, both relaxation rates indicate protein degradation for all three Abs, with the observed decreases in *R*_2_ specifically indicating fragmentation occurring in all three Abs. Therefore, for COE-03, the observation of fragmentation based on relaxation rates, yet no net change in 1D spectra, infers that aggregation must also be occurring for this mAb. For COE-07 and COE-19, the NMR observables show the predominant occurrence of fragmentation but do not rule out aggregation in these two Abs.

### Protein Degradation Detected by High-Performance Size Exclusion Chromatography Analysis

**Figure 5:**
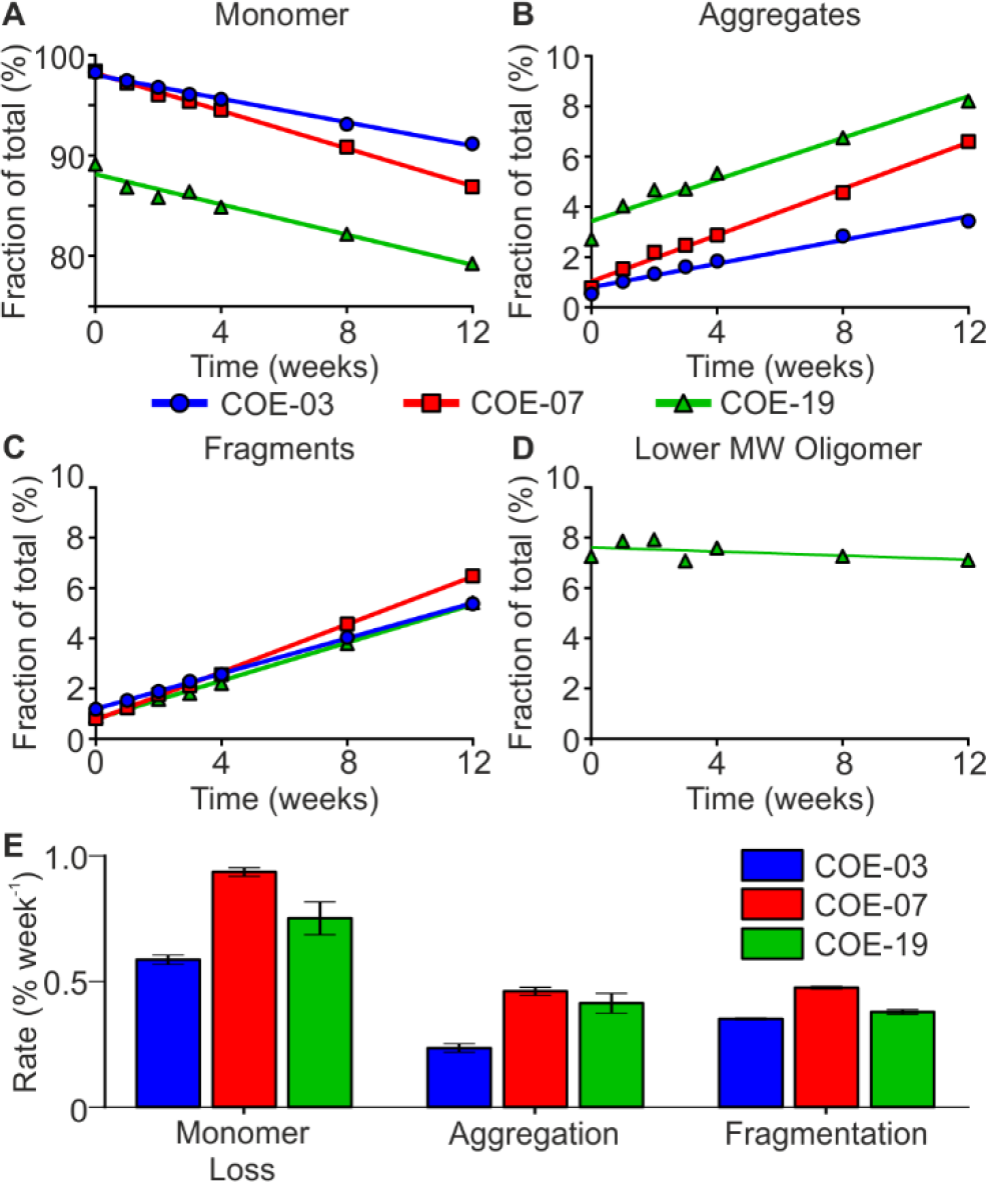
Assessment of monomer, aggregate, and fragment content by HPSEC. Monomer (A), aggregate (B), and fragment (C) species as a percentage of total observed species. (D) Lower molecular weight oligomer detected in COE-19. Experimental data with linear fit. (E) Rates of monomer loss, aggregation, and fragmentation per week, derived from linear fits with 95% confidence intervals.

To relate NMR observations of Ab stability with standard orthogonal measurements, we analyzed the monomer, aggregate, and fragment content using high performance size exclusion chromatography (HPSEC) (see Figure S3 for chromatograms) of Ab samples stored in identical formulations under identical conditions (Figure 5). All three Abs exhibited both fragmentation and aggregation in a time-dependent manner, with the rates of aggregation and fragmentation slowest for COE-03. Additionally, a lower molecular weight oligomer and a higher initial level of aggregates were detected in COE-19 samples (Figure 5D). This concurs with lower observed initial 1D NMR signal intensity for COE-19, with these oligomers expected to contribute less to the observable signal (Figure 1). Overall, the NMR observations of protein degradation are in agreement with the HPSEC measurements, with NMR spectra sensitive to protein degradation occurring at a rate of <1% per week.

### Small Molecule Degradation Detected by T_2_-Filtered ^1^H NMR

**Figure 6:**
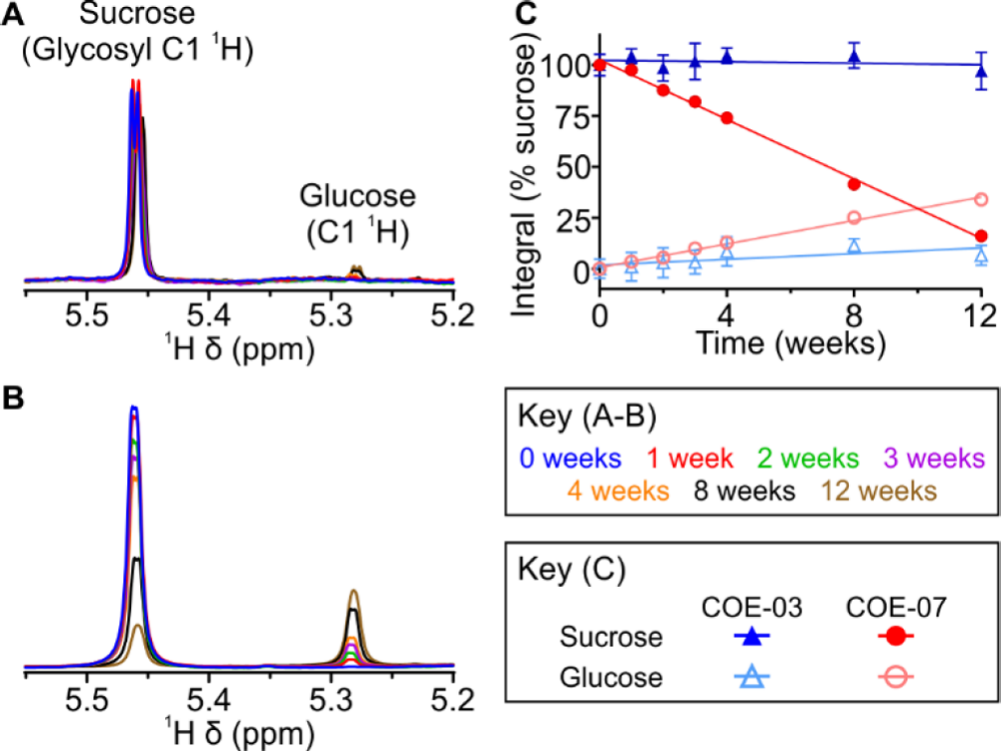
Degradation of sucrose detected by ^1^H NMR spectroscopy. ^1^H NMR spectra for (A) COE-03 and (B) COE-07, with sucrose (glycosyl C1-^1^H) and glucose (C1-^1^H) at 5.46 and 5.28 ppm, respectively. (C) Change in sucrose and glucose integrals (expressed as a percentage of the initial sucrose integral in each Ab sample) over time. Mean ± SD for three replicates, with linear fit.

Along with stability of the biopharmaceutical protein itself, the stability of small molecule components such as buffers and excipients is critical to the overall formulation. With this in mind, the NMR signals from the residual small molecule components from the original Ab formulations were monitored using T_2_-filtered experiments (116 ms filter), which essentially remove signals from the faster relaxing protein. Over time a number of small molecules exhibited changes in NMR signals associated with degradation. In COE-03 and COE-07 samples, sucrose (not present in COE-19) exhibited reduction in glycosyl C1-^1^H signal intensity, accompanied by appearance and increases in glucose C1-^1^H signal (Figure 6A,B). This degradation was markedly greater in COE-07 than in COE-03 (Figure 6C). Notably in COE-07 samples, increases in glucose signal were not proportional with sucrose signal reduction, as would be expected from the breakdown of one sucrose molecule into one molecule of glucose and one molecule of fructose. This indicates further degradation of glucose in COE-07, potentially in the form of glycation of protein molecules.

**Figure 7:**
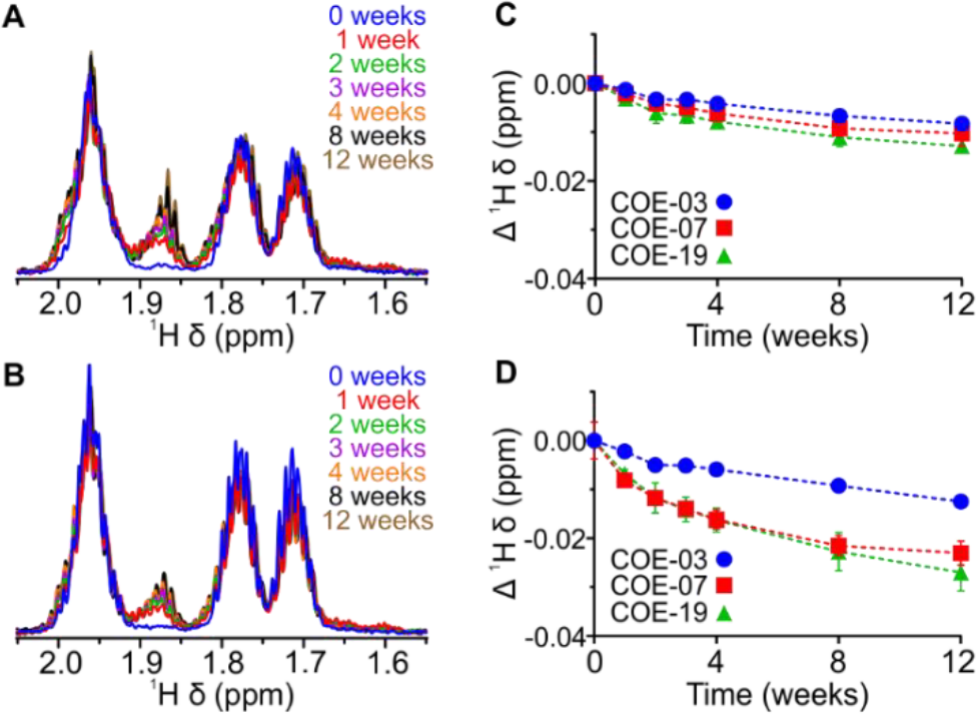
Arginine degradation and solution pH changes detected by ^1^H NMR spectroscopy. ^1^H NMR spectral region containing arginine signals (Cβ- and Cγ-^1^H’s) for (A) COE-07 and (B) COE-19. Changes (Δ) in ^1^H chemical shifts for histidine C_δ2_-^1^H (C) and C_ε1_-^1^H (D) over the course of the stability study. Mean ± SD for three replicates, with dashed lines as guides only.

^1^H NMR also detected degradation of arginine present in COE-07 and COE-19 formulations (not present in COE-03) (Figure 7A,B). Here, reduction in arginine signals was accompanied by the appearance of new upfield resonances, consistent with the arginine oxidation.^36^ Arginine degradation occurred at similar levels in both Ab solutions. Finally, histidine present in all Ab samples exhibited minor upfield shifts in both imidazole carbon bound protons (Figure 7C,D). These signals are sensitive to solution pH as a result of imidazole ring protonation, and as such these changes suggest a slight increase (∼0.1 pH units based on calibration curves^37^) in solution pH over the 12-week period. All together the data suggests that comprehensive analysis of even a simple set of NMR spectra, including ^1^H 1D, supplemented by *R*_1_ and *R*_2_ relaxation profiles and T_2_-filtered 1D experiments, can provide a comprehensive assessment of protein formulations and reveal degradation of both protein and small molecules.

## Discussion

Monitoring both small molecule and protein content and stability is vital to the successful optimization of Ab formulations and achieving a long product shelf life. However, assessment of formulations typically requires numerous techniques, with separate sample manipulations which may distort the analysis; for example, sample dilution would alter the equilibrium of reversible self-association present in the original formulation. We show here that ^1^H NMR spectroscopy can be used as an orthogonal technique to simultaneously characterize the content and stability of both protein and small molecule formulation components, perhaps as a triaging approach to inform decisions on which specialized techniques should be employed to quantify or study particular forms of degradation in more detail.

In NMR, initial Ab signal intensity and relaxation rates report on Ab solution behavior, with molecules exhibiting greater self-association or with oligomer species displaying lower signal intensities and higher relaxation rates (Figure 1). Following degradation at 40 °C, the observed broad increases in Ab signal intensity with retention of the overall spectral appearance (Figure 2) indicate fragmentation in COE-07 and COE-19. This is consistent with previous comparisons of enzymatically digested mAb fragments with intact mAbs by 2D natural abundance NMR.^27^ Conversely, 1D spectra may exhibit reductions in intensity if aggregation is dominant.^35^ However, as 1D ^1^H NMR spectra represent a balance between monomers, aggregates, and fragments, such spectra coincidentally may be insensitive to degradation if the effects of aggregation and fragmentation on signal intensity cancel each other out (Figure S2), as in the case of COE-03 here (Figures 2 and 5). However, the addition of relaxation rate analysis revealed that fragmentation occurred in COE-03 (Figures 3 and 4), which, with the observed static 1D spectra, was allowed to infer the occurrence of aggregation. For the two other Abs, 1D spectral changes revealed the presence of significant degradation immediately. As protein and small molecule signals are monitored simultaneously, this more detailed approach may be advantageous compared to recently suggested analysis based on the single parameter relaxation rate of the water signal.^33,38^ Our approach could also be extended to study chemical modification of Abs^39,40^ and small molecule formulation components at the same time, alongside Ab degradation.

Small molecules, such as buffers and excipients, are also an integral component of biopharmaceutical formulations, responsible for stabilizing and solubilizing the therapeutic protein. If they degrade, their stabilizing function may be diminished. Most common buffers and excipients contain NMR observable protons.^31^ As demonstrated here, ^1^H NMR is well suited to monitoring the presence and degradation of small molecules, particularly after the application of a T_2_ filter to remove fast relaxing protein signals (Figures 6 and 7). This is particularly applicable to studying the degradation of sacrificial excipients, such as methionine^41,42^ and other antioxidants, which are believed to protect proteins from degradation by undergoing degradation themselves. Additionally, the chemical shift of ionizable species, such as buffer molecules (e.g., histidine here) or spiked into solution as a tracer, can be used as an inbuilt pH meter (Figure 7) when compared to a known calibration curve. These assessments of small molecule stability may be coupled with the NMR identification of small molecule contaminants or processing impurities^29,30^ to provide an overarching assessment of the small molecule content of solutions throughout the manufacturing process.

## Conclusion

NMR assessment of both protein and small molecule components provides a holistic characterization of the content and stability of an overall biopharmaceutical formulation. Observation of changes in protein signal intensity or apparent relaxation rates indicate that monomer, aggregate, and fragment content should be investigated, for example, by HPSEC or capillary gel electrophoresis (CGE). Changes in excipient signal intensity or chemical shift or appearance of new signals indicates chemical degradation of small molecules. After detection of degradation of specific small molecules, such as sucrose degradation into glucose observed here, specific protein modifications, such as protein glycation which may impact pharmacokinetics and pharmacodynamics,^43,44^ should be investigated. This NMR assessment of small molecule and protein content and stability can be performed *in situ* at high concentration without further sample manipulation, making it a useful orthogonal assessment of overall formulation stability and helping to triage the use of specialized techniques for more detailed characterization.

## Supporting Information Available

Figure S1, T_2_-filtered NMR spectra at *T* = 0; Figure S2, illustrative modeling of effect of Ab aggregation and fragmentation on NMR signal intensity; and Figure S3, HPSEC chromatograms showing Ab degradation (PDF)
