# Supplementary material for: Comprehensive Assessment of Protein and Excipient Stability in Biopharmaceutical Formulations Using 1H NMR Spectroscopy
Source: ACS Pharmacol Transl Sci. 2020 Dec 16;4(1):288–95. doi: 10.1021/acsptsci.0c00188 (PMC7906489; doi:10.1021/acsptsci.0c00188)
Supplement: Supplementary file 1 — pt0c00188_si_001.pdf [file pt0c00188_si_001.pdf]

SUPPORTING INFORMATION

Comprehensive assessment of protein and excipient stability in biopharmaceutical formulations using ^{1}H NMR spectroscopy

Jack E. Bramham[1], Adrian Podmore[2†], Stephanie A. Davies[2], and Alexander P. Golovanov[1*]

[1] Manchester Institute of Biotechnology and School of Chemistry, Faculty of Science and Engineering, The University of Manchester, Manchester, M1 7DN, UK

[2] Dosage Form Design & Development, BioPharmaceuticals Development, R&D, AstraZeneca, Cambridge, CB21 6GH, UK

Corresponding Author

* E-mail: a.golovanov@manchester.ac.uk

Present Addresses

† Arecor Limited, Chesterford Research Park, Little Chesterford, CB10 1XL

Table of Contents:

Figure S1: T2-filtered NMR spectra of the three Ab formulations at T=0, highlighting the presence of the small molecule components. (Page S-2)

Figure S2: Modelling of the effects of aggregation and fragmentation, in isolation and together, on 1D ^{1}H NMR signal intensity. (Page S-3)

Figure S3: HPSEC chromatograms of the three Abs over 12 weeks accelerated stability storage. (Page S-4)

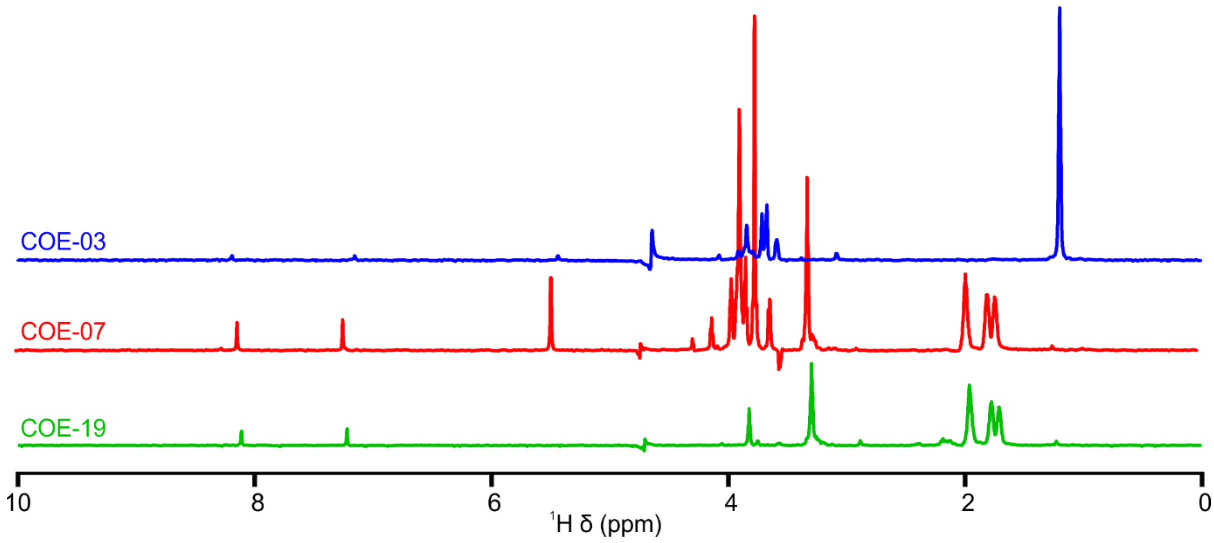

Figure S1. T_2-filtered NMR spectra of the three Ab formulations at T=0, highlighting the presence of the small molecule components. A 116 ms T_2 filter was used, corresponding to 32 echoes with 3.2 ms delay. COE-03 (blue), COE-07 (red), and COE-19 (green). All spectra were acquired with the same receiver gain, 24 scans, and for samples with same Ab concentration, and as such the signal intensities reflect the levels of small molecule constituents present in solution after extensive dialysis.

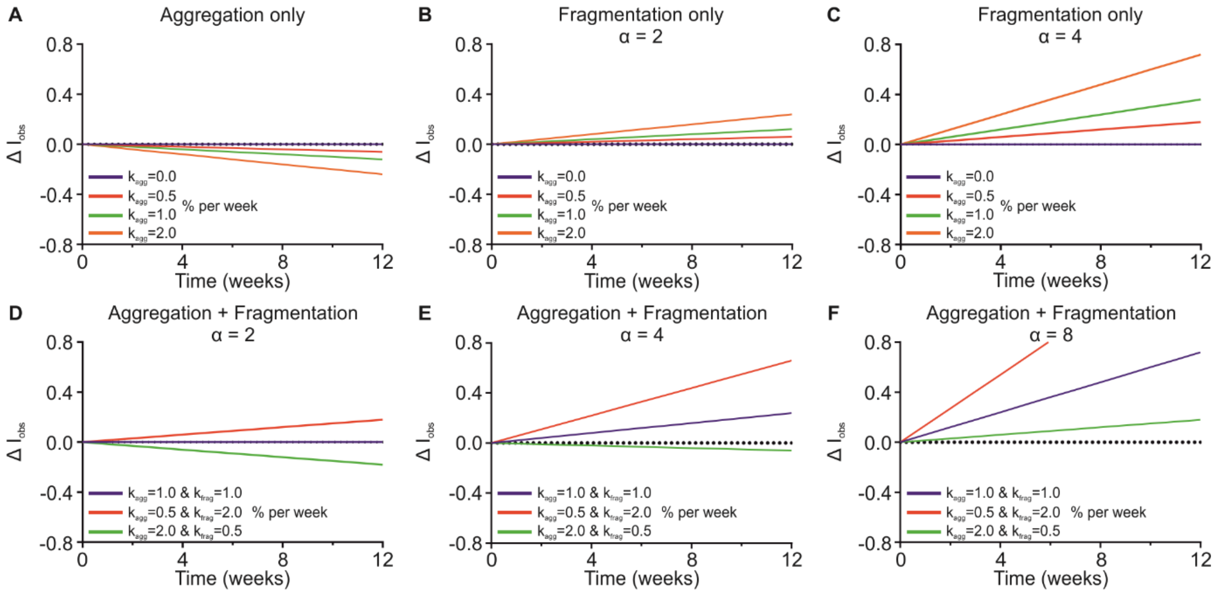

Figure S2. Modelling of the effects of aggregation and fragmentation, in isolation and together, on 1D ^{1}H NMR signal intensity. (**A**) Aggregation only and (**B&C**) fragmentation only, with α=2 and α=4, respectively. Combination of aggregation and fragmentation, with (**D**) α=2, (**E**) α=4, and (**F**) α=8. Change in observed signal intensity (ΔI_{obs}) from the initial value modelled against storage time. For a Ab formulation, I_{obs} can be described as:

$$I_{obs} = (P_{mon} \times I_{mon}) + \left(P_{frag} \times I_{frag}\right) + (P_{agg} \times I_{agg}) \quad (1)$$

where I_{mon}, I_{frag} and I_{agg} are the characteristic signal intensities of monomer, fragment and aggregate species, respectively, for a given protein/solution conditions, and P_{mon}, P_{frag} and P_{agg} are the populations of each species. These populations are time-dependent, with $P_{mon} = P_{mon,0} + k_{mon} \times t$, $P_{frag} = k_{frag} \times t$ and $P_{agg} = k_{agg} \times t$, where k_{mon}, k_{frag} and k_{agg} are the rates (% per week) of monomer loss, and fragmentation, and aggregation, respectively; t is time, and $P_{mon,0}$ is initial population of the monomer. The populations are normalised as: $P_{mon} + P_{frag} + P_{agg} = 1$. For fragments which tumble faster than monomer we assume $I_{frag} = \alpha\, I_{mon}$, where α>1 (and may vary between Abs or solution conditions). For large aggregates such as those generated by Abs, we assume $I_{agg} \approx 0$. Although this simple model is clearly underdetermined and does not allow quantitative fitting of experimental parameters, it can be used to illustrate some typical behaviours of this system in different regimes. Aggregation alone (A) results in decreases in signal intensity, whilst fragmentation alone (B&C) results in increases in signal intensity, with the extent of the increase dependent upon the value of α. For systems undergoing both aggregation and fragmentation (D-F), in certain situations there may be no or negligible change in I_{obs} if the effects of aggregation and fragmentation counteract each other.

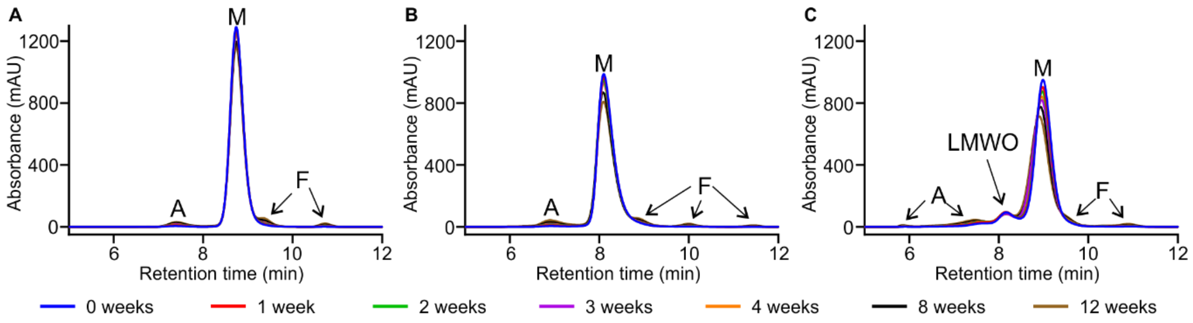

Figure S3. HPSEC chromatograms of the three Abs over 12 weeks accelerated stability storage. (A) COE-03, (B) COE-07, and (C) COE-19 chromatograms. Aggregates are marked with 'A', monomer peaks with 'M', and fragments with 'F'. The lower molecular weight oligomer species in COE-19 is marked 'LMWO'.
